# Peer Review of "Exercise-Induced Hypoalgesia Following Proprioceptive Neuromuscular Facilitation and Resistance Training Among Individuals With Shoulder Myofascial Pain: Randomized Controlled Trial"

**DOI:** 10.2196/45034

**Published:** 2022-12-27

**Authors:** Jonathan Greenberg

**Affiliations:** 1 Department of Psychiatry Harvard Medical School Boston, MA United States; 2 Massachusetts General Hospital Boston, MA United States

**Keywords:** exercise induced hypoalgesia, proprioceptive neuromuscular facilitation, PNF, resistance exercise, conditioned pain modulation, myofascial pain syndrome, resistance training, hypoalgesia, exercise-induced hypoalgesia, shoulder myofascial pain, myofascial pain, pain management, chronic pain, musculoskeletal pain, physical therapy, physiotherapy, shoulder pain, upper back pain, exercise, pain

*This is a peer-review report submitted for the paper “Exercise-Induced Hypoalgesia Following Proprioceptive Neuromuscular Facilitation and Resistance Training Among Individuals With Shoulder Myofascial Pain: Randomized Controlled Trial”*

## Round 1 Review

This paper [1] set to estimate the effect of proprioceptive neuromuscular facilitation (PNF) and resistance training on exercise-induced analgesia and conditioned pain modulation among young adult women with myofascial pain syndrome.

The paper holds several strengths, including random assignment and the inclusion of PNF, 2 resistance training exercise types, and 1 passive control group, which enables comparison across exercise conditions. Authors justifiably correct for multiple comparisons. The discussion thoroughly interprets the findings and relates them to existing literature. Some questions and potential limitations are listed below.

### Specific Comments

#### Major Comments

1. The study is limited to young women (18-30 years old) and therefore has limited generalizability to men, as well as women above the age of 30 years. Authors partially acknowledge this in the limitations section (with regard to gender).

2. The sample size in each group is modest (n=18-20), limiting statistical power.

3. Did the authors have a specific hypothesis about the relative effect of PNF, isometric, and isotonic exercise training on outcomes? Such a hypothesis is now stated. Was the testing of differential effects exploratory?

4. Authors indicate that “Randomized sequences were generated by computer.” Can authors provide details on the method, software, or website used for randomization?

5. Authors indicate that participants were excluded if they experienced depression, psychosis, cognitive impairment, etc. How were these assessed?

#### Minor Comments

6. Authors make use of 6 different acronyms in the abstract, which may make it more difficult to read, particularly for individuals outside this immediate field. When possible, consider spelling things out to increase ease of readability.

7. Please change all instances of “*P*<.000” to “*P*<.001”

8. There are some typos throughout the manuscript, please correct those (eg, “Our findings mostly met what we previously hypothesized, where was an increase in PPT at trigger point”; which may have been “There was an increase,” or “Crombie et al investigated that the serum endocannabinoids increased,” which may have been “Crombie et al ‘found’ or ‘reported’ that….,” as well as other examples throughout).

## Round 2 Review

Thank you for revising the manuscript. The authors have addressed my concerns.

## Abbreviations

PNF: proprioceptive neuromuscular facilitation
